# T-cell-based immunotherapy of acute myeloid leukemia: current concepts and future developments

**DOI:** 10.1038/s41375-021-01253-x

**Published:** 2021-05-05

**Authors:** Naval Daver, Ahmad S. Alotaibi, Veit Bücklein, Marion Subklewe

**Affiliations:** 1 Department of Leukemia, MD Anderson Cancer Center, Houston, TX USA; 2 Oncology Center, King Faisal Specialist Hospital and Research Center, Riyadh, Saudi Arabia; 3 Department of Medicine III, University Hospital, LMU Munich, Munich, Germany; 4 Laboratory for Translational Cancer Immunology, LMU Gene Center, Munich, Germany; 5 German Cancer Consortium (DKTK), German Cancer Research Center (DKFZ), Heidelberg, Germany

**Keywords:** Diseases, Medical research

## Abstract

Acute myeloid leukemia (AML) is a heterogeneous disease linked to a broad spectrum of molecular alterations, and as such, long-term disease control requires multiple therapeutic approaches. Driven largely by an improved understanding and targeting of these molecular aberrations, AML treatment has rapidly evolved over the last 3–5 years. The stellar successes of immunotherapies that harness the power of T cells to treat solid tumors and an improved understanding of the immune systems of patients with hematologic malignancies have led to major efforts to develop immunotherapies for the treatment of patients with AML. Several immunotherapies that harness T cells against AML are in various stages of preclinical and clinical development. These include bispecific and dual antigen receptor-targeting antibodies (targeted to CD33, CD123, CLL-1, and others), chimeric antigen receptor (CAR) T-cell therapies, and T-cell immune checkpoint inhibitors (including those targeting PD-1, PD-L1, CTLA-4, and newer targets such as TIM3 and STING). The current and future directions of these T-cell-based immunotherapies in the treatment landscape of AML are discussed in this review.

## Introduction

For decades, immunotherapy—in the form of allogeneic hematopoietic stem cell transplantation (allo-HSCT)—has been a cornerstone of the treatment of acute myeloid leukemia (AML) and other hematologic malignancies, offering the potential to cure a subset of patients. T cells are considered the major contributors to the success of this therapy, as demonstrated, for example, by the efficacy of donor lymphocyte infusions to eradicate residual disease after transplantation [1]. However, allo-HSCT has major limitations owing to significant, often long-term, side effects. T cells can, apart from inducing a desirable graft-versus-leukemia effect, also mediate harmful graft-versus-host disease (GvHD). Innovative T-cell-based treatment strategies aim to achieve robust antileukemic activity while avoiding T-cell cytotoxicity against healthy tissues.

In recent decades, three different treatment platforms have been developed to harness antineoplastic T-cell activity:

1. Recruitment of T cells independently of T-cell receptor (TCR) specificity through T-cell-engaging antibody constructs, and
2. Genetic engineering of T cells [TCR-modified and chimeric antigen receptor (CAR) T cells].
3. Reactivation of endogenous T-cell responses through immune checkpoint inhibitors.

These platforms have been successfully implemented against hematologic malignancies—to date, mainly in B-cell neoplasias. Blinatumomab, a bispecific T-cell engager (BiTE), has been used to treat B-cell precursor acute lymphoblastic leukemia (BCP-ALL) [2, 3]. CAR-T cells induce high remission rates in heavily pretreated BCP-ALL [4], diffuse large B-cell lymphoma (DLBCL), and primary mediastinal B-cell lymphoma (PMBCL) patients [5, 6]. In addition, immune checkpoint inhibitors (ICPIs) have been approved for the treatment of Hodgkin’s lymphoma and PMBCL [7–9].

However, the translation of these successes into treatments for AML has been challenging owing to the lack of suitable target antigens. Here, we review the current data, discuss immunotherapeutic treatment strategies, delineate the potential paths forward to successful implementation, and propose the use of biomarker-driven clinical studies for the development of individualized treatment approaches.

## Target antigens in T-cell-based immunotherapy

Ideally, any target antigen for antibody- or CAR-T cell-based AML immunotherapy effectively identifies neoplastic cells and spares healthy tissue. To achieve these goals, an ideal AML target should be (1) expressed (strongly) on the surface of the AML blasts, (2) expressed in the majority of AML cases, but (3) should not be expressed in healthy bone marrow or extramedullary cells. In addition, optimal target antigens are expressed on leukemic stem cells (LSCs) and progenitor cells, a subpopulation of AML cells with self-renewal, and chemorefractory capacity.

The identification of antigens that incorporate all these properties has been challenging. Although expression intensity can be higher in AML bulk cells and/or LSCs, target-antigen candidates such as CD33 and CD123 are frequently found on hematopoietic stem cells (HSCs), resulting in the risk of long-lasting or even permanent myelosuppression [10]. Results from clinical trials targeting alternative antigens that are known not to be expressed on HSCs (e.g., CD44v6 or TIM3) are yet to be published.

## Classification of target antigens

### Leukemia-specific antigens

Leukemia-specific neoantigens, resulting from (ideally leukemogenic) mutations, are usually expressed intracellularly and are presented in the context of HLA molecules (Table 1). As these antigens result from aberrant proteins encoded by leukemia mutations, they are exclusively expressed in malignant clones and therefore might represent “ideal” targets. However, not all these intracellular antigens are presented on the cell surface (as evidenced by the lack of spontaneous T-cell responses against, e.g., DEK–CAN fusion proteins). Leukemia-specific neoantigens have not been evaluated in AML clinical trials to date.

**Table 1:** Leukemia-specific target antigens in AML.

Target antigen	Expression	Spontaneous immune responses	Ref.
Mutated NPM1	Intracellular	CD8+ T-cell responses observed in AML; might contribute to favorable outcome of NPM1^mut^ AML	Greiner et al. [86], van der Lee et al. [87]
IDH1^R132H^	Intracellular	CD4+ T-cell responses observed in glioma	Schumacher et al. [88]
Mutated FLT3 (ITD)	Intracellular	CD8+ T-cell responses observed in AML	Graf et al. [89]
PML-RARA	Intracellular	None observed	Gambacorti-Passerini et al. [90]
DEK–CAN	Intracellular	None observed	Makita et al. [91]

### Lineage-restricted antigens

Lineage-restricted antigens are usually cell-surface antigens confined to the myeloid lineage. The majority of current clinical trials of antibody constructs or CAR-T cells in AML patients target lineage-restricted antigens, most commonly CD33 and CD123 (Table 2).

**Table 2:** Lineage-restricted target antigens in AML.

Target antigen	Expression	Physiological function	Expression on bulk AML cells	Expression on LSCs	Expression on HSCs	Nonhematopoietic expression	Spontaneous immune responses	Clinical trials in AML?	Ref.
CD33	Surface	Sialic-acid-dependent cytoadhesion molecule	>99% (higher expression with normal karyotype or e.g., NPM1+)	+	+	Kupffer cells (liver), microglial cells (CNS)		ADC, BiTE, CAR-T	Krupka et al. [24]
CD123	Surface	Interleukin 3 receptor	~78% (higher expression in FLT3-ITD-mutated AML)	++	(+)	Bronchus/gastrointestinal tissue		CAR-T, DART	Haubner et al. [10], Kovtun et al. [92], Ehninger et al. [93]
CLL-1/CLEC12A	Surface	Inhibitory C-type lectin-like receptor involved in immunological homeostasis	78–92% (lower expression in adverse risk cytogenetics)	+	−	N.r.		CAR-T (in combination with CD33), IgG1 bispecific antibody	Morsink et al. [39], Wang et al. [40], van Rhenen et al. [41]
CD117	Surface	Mast/stem cell growth factor receptor	78–90%	(−)	+	Epithelial cells (e.g., in skin, breast tissue), Cajal cells, melanocytes		CAR-T	Escribano et al. [94], Scolnik et al. [95]
CD135/FLT3	Surface	Cytokine receptor	54–90%	++	(+)	CNS, intestine, testis (no surface expression)		BiTE	Brauchle et al. [43], Kandeel et al. [96]
Folate receptor β	Surface	Folate uptake	~70%	N.a.	(+)	N.r.		−	Lynn et al. [97]
IL1RAP	Surface	IL1 receptor accessory protein	~80%	+	(−)	Esophagus		−	Mitchell et al. [98], Askmyr et al. [99]
PR1/proteinase-3-derived epitope peptide	Intracellular	Neutrophilic serine proteases	+	+	+	−	CD8+ T-cell responses observed in AML	Vaccination trials	Sergeeva et al. [100], Alatrash et al. [101]

Antigen expression: − negative, (+) low, + positive, ++ highly expressed (−) infrequent.

*ADC* antibody–drug conjugate, *BiTE* bispecific T-cell engager, *CAR T* chimeric antigen receptor T cells, *CNS* central nervous system, *DART* dual-affinity retargeting antibody, *HSC* hematopoietic stem cell, *LSC* leukemic stem cell, *NK cell* natural killer cell, *n.a.* not assessed, *n.r.* not reported.

### Leukemia-associated antigens

Leukemia-associated antigens are overexpressed on AML cells relative to healthy tissue and are usually not lineage specific, making expression on healthy hematopoietic cells (and thereby HSC toxicity and subsequent aplasia) less likely (Table 3). However, these antigens may be found on nonhematopoietic tissues, resulting in on-target off-tumor toxicities. WT1 and PRAME are being evaluated in early-phase clinical trials in patients with AML [11–13]. Strategies to identify additional antigens that are exclusively expressed on AML cells (including LSCs) includes comparing transcriptome and surfaceome data of AML cell lines, primary AML cells and healthy hematopoietic cells. Using this approach, several promising candidates have been identified [14, 15].

**Table 3:** Leukemia-associated target antigens in AML.

Target antigen	Expression	Physiological function	Expression on bulk AML cells	Expression on LSCs	Expression on HSCs	Nonhematopoietic expression	Spontaneous immune responses	Clinical trials in AML?	Ref.
Lewis Y (CD174)	Surface	Unknown	+	N.a.	(−)	Epithelial cells		CAR-T	Muroi et al. [102], Zhang et al. [103]
MUC1	Surface	Mucosal protection	+ (Myelomonocytic/monocytic AMLs)	+	(−)	Epithelial cells		CAR-T	Stroopinsky et al. [104, 105]
CD44v6	Surface	Cell–cell interactions/cell–matrix interactions	+ (64–72%)	Probable	−	Keratinocytes		CAR-T	Legras et al. [106], Neu et al. [107], Casucci et al. [108]
CD244/2B4	Surface	Activating/inhibitory receptor of NK cells	++	++	++	–		−	Haubner et al. [10]
CD96	Surface	Immune cell adhesion	+	+	(−)	−		−	Hosen et al. [109]
TIM-3	Surface	Co-inhibitory receptor of immune cells	+ (87%)	+ (78%)	−	Bladder?		Antibody (checkpoint inhibition)	Haubner et al. [10], He et al. [52], Kikushige et al. [110]
CD70	Surface	Ligand of CD27 involved in immune cell homeostasis	+ (>95%)	+	−	Medullary thymic epithelial cells		Fc-engineered antibody	Riether et al. [111]
WT1/Wilms’ tumor gene 1	Intracellular	Transcription factor	+ (73–100%)	+	+	Kidney, spleen, heart, lung, prostate	CD8+ T-cell responses observed in AML	Vaccination trials, TCR-transgenic T cells	Tawara et al. [11], Lichtenegger et al. [13], Rosenfeld et al. [112]
PRAME	Intracellular	(Cancer testis antigen)	+ (41–55%)	+	Minimal	Testis	CD8+ T-cell responses observed in AML	Vaccination trials	Qin et al. [113], Ding et al. [114], Rezvani et al. [115]
RHAMM	Intracellular	Cell–matrix interactions	+	Questioned	+	Colon	CD8+ T-cell responses rarely observed in AML	Vaccination trials	Casalegno-Garduño et al. [116], Greiner et al. [117], Snauwaert et al. [118]
Survivin	Intracellular	Anti-apoptotic protein (relevance in embryogenesis)	+	+	+	Endothelial cells	T-cell responses observed in breast cancer, melanoma, and CLL	Vaccination trials	Andersen et al. [119], Carter et al. [120], Xing et al. [121], Fukuda et al. [122]
hTERT	Intracellular	Subunit of the telomerase complex	+	Questioned	(+)	Keratinocytes, testis, endometrium, placenta	CD8+ T-cell responses rarely observed in HCC or MM	Vaccination trials	Hiyama et al. [123], Bruedigam et al. [124], Hartman et al. [125]

Antigen expression: − negative, (+) low, + positive, ++ highly expressed (−) infrequent.

*CAR-T* chimeric antigen receptor T cells, *HCC* hepatocellular carcinoma, *HSC* hematopoietic stem cell, *LSC* leukemic stem cell, *MM* multiple myeloma, *n.a.* not assessed.

## Combinatorial approaches

So far, a single target antigen as ideal as CD19 or CD22 in B-cell ALLs has not been identified for AML. Combination strategies, in which several different target antigens are used to target AML cells and LSCs, might increase specificity. Such multitargeting approaches might also reduce the risk of target-antigen downregulation on malignant cells, an escape mechanism frequently observed in patients after anti-CD19 CAR-T-cell therapy [16, 17]. Combinatorial targeting of different AML target antigens might be used in parallel (e.g., by simultaneous use of two or more T-cell-recruiting antibodies with different target-antigen specificity, or with dual CAR-T cell approaches) or sequentially (e.g., by consecutive infusion of antibodies or CAR-T cells with different target-antigen specificity). Optimal treatment sequences might be patient-specific, and remain to be elucidated.

## Selection of a target antigen

Three characteristics related to the expression of the antigen are of importance when evaluating it as a target for immunotherapy.

### Localization

HLA-restricted antigens are expressed intracellularly and can only be targeted with receptors that recognize this antigen in the context of a presenting HLA molecule (e.g., by TCR-modified T cells transduced with a full synthetic TCR, or by T-cell bispecific antibodies). In contrast, HLA-unrestricted antigens are expressed on the cell surface and are accessible to, for example, CARs.

### Expression intensity

Target antigens can be expressed with very low intensity on cells and, in such cases, might be undetectable by even sensitive methods such as flow cytometry. Nonetheless, dim expression might be sufficient to direct CAR-T cells against these cells, as demonstrated for anti-CD19 CAR-T cells in multiple myeloma samples [10].

### Expression distribution

The pattern of target-antigen expression might influence the pharmacokinetics of, for example, antibody constructs. Interestingly, in a phase I clinical trial, the applied dosage of the CD33-targeting BiTE AMG 330 was significantly higher than that used of blinatumomab, its CD19-targeting equivalent. In comparison to the strictly B-lymphocyte-specific expression of CD19, the wider expression of CD33 on different cell types likely causes large amounts of the BiTE to bind to off-tumor sites. This not only poses a risk of on-target off-tumor toxicity, but might also influence the biologically active half-life of the molecule by creating an “antigen sink” effect. Interindividual differences of an antigen sink might therefore influence the efficacy and toxicity of a targeted molecule.

## Recruitment of T cells independent of TCR specificity

### T-cell-recruiting antibody constructs: BiTEs, DARTs, and others

Bispecific antibodies are recombinant proteins that recruit T cells, through CD3 engagement, and target tumor cells, usually with a higher affinity, through binding to a tumor-associated antigen. Blinatumomab (a BiTE antibody) is the only bispecific antibody approved by both the US Food and Drug Administration (FDA) and the European Medicines Agency (EMA). Blinatumomab binds CD19 on B cells and CD3 on T cells and is used to treat patients with relapsed/refractory (R/R) or minimal residual disease (MRD)-positive (≥10^−3^) BCP-ALL [2, 3]. Different formats of bispecific antibodies have been developed, such as BiTEs, half-life-extended BiTEs, dual-affinity retargeting (DART) antibodies, tandem diabodies, DuoBody antibodies, affinity-tailored adaptors for T cells, and tetravalent bispecific antibodies. Some of these formats were designed for practical reasons related to construction and manufacturing, whereas others were designed with their biological characteristics, including pharmacokinetics, in mind [18, 19].

A major challenge in translating the success of bispecific antibody constructs from B-cell neoplasias to AML has been the identification of suitable target antigens. As discussed in the section “Target antigens in T-cell-based immunotherapy”, several AML-selective antigens are being investigated as therapeutic targets. Among these, bispecific antibodies targeting the lineage-restricted antigens CD33, CD123, CLL-1 (CLEC12A), and FLT3 are in early clinical trials and are discussed below.

#### Targeting CD33 with T-cell-recruiting antibody constructs

CD33 is widely expressed in human AML cells. CD33’s validity as a therapeutic target in AML was exemplified by gemtuzumab ozogamicin—an antibody–drug conjugate (ADC) directed against CD33—which, in combination with daunorubicin and cytarabine, was approved by both the US FDA and the EMA for the treatment of newly diagnosed CD33-positive AML [20–23]. Several CD33 × CD3 bispecific antibodies are in ongoing clinical trials.

AMG 330, a BiTE molecule [24] was able to kill AML cells in primary human AML samples across a wide range of effector:target (E:T) ratios in ex vivo experiments, and was able to continuously expand and activate T cells [24]. It is currently being tested in a phase I trial in adult R/R AML patients, given as a continuous intravenous infusion because of its short half-life (<2 h; NCT02520427). The updated results of this trial included 60 treated patients [25]. This trial used a dose-step approach together with dexamethasone prophylaxis in order to prevent cytokine release syndrome (CRS) and to achieve high targeted doses. CRS was the most commonly observed treatment-related adverse event (TRAE): 40 of the 60 treated patient (67%) developed CRS; reaching grade 3 or higher in nine patients (15% of the total). The CRS was mitigated through implementation of three dose-steps and early use of tocilizumab, an anti-IL-6 antibody, approved for CAR-T-cell-mediated CRS. Other commonly observed TRAEs were of lower grade and included skin disorders in 58%, elevated liver function tests in 25%, and gastrointestinal disorders in 30% of the patients. AMG 330 exposures and E:T ratios were positively correlated with CRS occurrence and severity. As expected, CRS frequency and severity were associated with the levels of IL-6 and IL-10 released upon treatment.

Seven patients achieved complete remission (CR), including four with incomplete hematologic recovery (CRi) and one morphologic leukemia-free state (MLFS). The minimal efficacious dose for achieving response was 120 µg/day, and the CR/CRi rate was 17% with doses ≥120 µg/day. The median duration of response was 58.5 days (range 14–121 days). Responders were more likely to have higher AMG 330 exposures and lower baseline leukemic burden, with no correlation between CD33 expression on AML blasts and response.

A major challenge in using AMG 330 is its short half-life, requiring continuous intravenous infusion. A logical development was the fusion of the N-terminus of a single-chain IgG Fc region to a CD33 x CD3 BiTE to create the half-life-extended molecule AMG 673. AMG 673 is currently in a phase I trial in adult patients with R/R AML (NCT03224819). In contrast to AMG 330, AMG 673 is administered as two 1 h intravenous infusions on days 1 and 5 during each 14-day cycle. As of March 23, 2020, 38 patients had been treated with 11 different doses of AMG 673, ranging from 0.05 to 110 μg per dose. CRS was reported in 63%, with 18% grade 3 or higher events. Of the 27 evaluable patients, five experienced ≥50% reduction of blasts in bone marrow, including one CRi [26, 27].

AMV564 is a bivalent CD33 x CD3 bispecific antibody. In a phase I clinical trial in adult patients with R/R AML, AMV564 is administered by continuous intravenous infusion for 14 consecutive days in 28-day cycles (NCT03144245). Thirty-six patients were treated with 10 dose levels using a lead-in dose-escalation schedule [28]. All 36 patients were evaluable for safety and no dose-limiting toxicity (DLT) was reported. The most common grade 3 or higher TRAE was anemia, observed in 11% of patients. Among 35 evaluable patients, one CR, one CRi, and one PR were reported. AMV564 was reported to have a terminal half-life of 2–3 days. Other CD33 x CD3 bispecific antibodies in clinical trials include GEM333 (NCT03516760) and JNJ-67571244 (NCT03915379), both in adult patients with R/R AML (Table 4).

**Table 4:** Targeting CD33: T-cell-recruiting bispecific antibodies.

Clinicaltrials.gov identifier	AML target antigen	Study name	Drug name	Combination therapy?	Clinical phase	Indication	Primary end point	Estimated enrollment	Estimated completion date	Sponsor	Country	Status
NCT02520427	CD33	A phase I first-in-human study evaluating the safety, tolerability, pharmacokinetics, pharmacodynamics, and efficacy of AMG 330 administered as continuous intravenous infusion	AMG 330	No	I	R/R AML	DLT, toxicity	50	2021	Amgen	USA, Germany, Netherlands	Recruiting
NCT03224819	CD33	Study of AMG 673 in subjects with R/R AML	AMG 673	No	I	R/R AML	DLT, toxicity	50	2022	Amgen	USA, Australia, Germany	Recruiting
NCT03516760	CD33	A multicenter, open-label, dose-escalating, phase I trial with GEM333, CD33-targeted bispecific antibody-engaging T cells	GEM333	No	I	R/R AML	MTD, DLT, toxicity	33	2019	GEMoab Monoclonals	Germany	Recruiting
NCT03144245	CD33	A phase I, first-in-human, open-label, dose-escalating study of AMV564, a CD33 x CD3 tandem diabody	AMV564	No	I	R/R AML	DLT, toxicity	50	2020	Amphivena	USA	Recruiting
NCT03915379	CD33	A study of JNJ-67571244 in participants with R/R AML or MDS	JNJ-67571244	No	I	R/R AML	DLT, toxicity, ORR	90	2021	Janssen Research and Development, LLC	USA, Germany, Spain	Recruiting
NCT03647800	CD33	Study of APVO436 in Patients with AML or MDS	APVO436	No	I	R/R AML	DLT	108	2020	Aptevo Research and Development LLC	USA	Recruiting

#### Targeting CD123 with T-cell recruiting antibody constructs

CD123, the IL-3 receptor alpha chain, is expressed in normal hematopoietic stem/progenitor cells (HSPCs) and myeloid cells but its expression is increased on AML blast and LSCs [29–32]. The CD123-based bispecific antibody that is most advanced in clinical development is flotetuzumab (MGD006), a CD123 x CD3 DART [33]. Flotetuzumab is being evaluated in an ongoing phase I/II clinical trial in patients ≥18 years old with primary induction failure (PIF) or early relapse (ER) AML (NCT02152956), and in patients up to 20 years old with R/R AML (NCT04158739). Data on 30 patients, 25 of whom had high-risk disease, treated with the recommended phase II dose of 500 ng/kg/day administered as a 7-day/week continuous infusion was presented at ASH 2018 [34]. Patients received a lead-in dose (30 ng/kg/day for 3 days, followed by 100 ng/kg/day for 4 days) during week 1, followed by 500 ng/kg/day during weeks 2–4 of cycle 1, and a 4 days on/3 days off schedule for cycle 2 and beyond. CRS occurred in all patients, including 13.3% at grade 3 or above, although most cases were transient and reversible. Among 27 response-evaluable patients, five achieved a CR/CRi. Intriguingly, four of 13 patients (31%) with primary chemotherapy refractory AML had CR/CRi, whereas none of the 11 patients with relapsed disease had CR/CRi [34]. In a follow-up report, 42 of 88 adults with R/R AML were treated with flotetuzumab in a dose-finding segment; the other 46 received the recommended phase 2 dose of 500 ng/kg/day [35]. Grade 1/2 CRS was the most common adverse event. Systematic application of stepwise dosing, pretreatment dexamethasone, early use of tociluzimab, and temporary dose interruptions helped to successfully prevent grade 3 or higher CRS. Thirty PIF/ER patients were treated at the recommended phase 2 dose, for whom the rate of CR/CR with partial hematological recovery (CRh) was 27% and the overall response rate (ORR) (CR/CRh/CRi) was 30%. The median overall survival (OS) among PIF/ER patients achieving CR/CRh was 10.2 months. In a related study, 442 primary bone-marrow samples from children and adults with AML were analyzed to identify immune-infiltrated and immune-depleted AML classes by applying gene and protein profiling [36]. Interestingly, interferon-gamma-related mRNA profiles were predictive for both chemotherapy resistance and response to flotetuzumab therapy, suggesting that this might be a potential biomarker for selecting AML patients most likely to benefit from flotetuzumab and potentially other similar immune-enhancing strategies [36].

Vibecotamab (XmAb 14045) is another CD123 x CD3 bispecific antibody in a phase I trial in patients with CD123-expressing hematological malignancies. The first results from 64 patients (63 with R/R AML, 1 with R/R B-ALL) presented at ASH 2019 [37] revealed no MTD but a DLT of grade 4 CRS at 2.3 µg/kg, leading to the recommended dose of 1.3 µg/kg. CRS was observed in 77%, including 11% with grade 3 or higher. Two CRs and one CRi were observed, all in patients treated with either the 1.3 or 2.3 µg/kg weekly dose, the two highest doses tested [37].

Other CD123 x CD3 bispecific antibodies in early-phase clinical trials in patients with R/R AML include SAR440334 (NCT03594955), a T-cell-engaging multispecific monoclonal antibody, APVO436 (NCT03647800), an optimized ADAPTIR bispecific antibody, and JNJ-63709178 (NCT02715011), a humanized DuoBody (Table 5). Results from these trials are yet to be reported.

**Table 5:** Targeting CD123 and other AML-associated target antigens: T-cell-recruiting bispecific antibodies.

Clinicaltrials.gov identifier	AML target antigen	Study name	Drug name	Combination therapy?	Clinical phase	Indication	Primary end point	Estimated enrollment	Estimated completion date	Sponsor	Country	Status
NCT02152956	CD123	Flotetuzumab in primary induction failure (PIF) or early relapse (ER) acute myeloid leukemia (AML)	Flotetuzumab (MGD006)	No	I	R/R AML, intermediate-2/high-risk MDS	DLT	124	2020	Macrogenics	USA, Germany, Netherlands	Recruiting
NCT04158739	CD123	Flotetuzumab and cytarabine for the treatment of R/R AML	Flotetuzumab (MGD006)	Yes, with cytarabine	I	R/R AML (children, adolescents, and young adults)	DLT, toxicity	47	2021	Macrogenics	USA, Australia, Germany	Recruiting
NCT02715011	CD123	A phase I, first-in-human, open-label, dose-escalation study of JNJ-63709178, a humanized CD123×CD3 DuoBody	JNJ-63709178	No	I	R/R AML	DLT, toxicity	60	2020	Janssen Research & Development	Germany	Recruiting
NCT02730312	CD123	A phase I multiple-dose study to evaluate the safety and tolerability of XmAb 14045 in patients with CD123-expressing hematologic malignancies	Xmab 14045	No	I	Primary or secondary AML	MTD, toxicity	66	2019	Xencor	USA	Recruiting
NCT03594955	CD123	First-in-human testing of dose-escalation of SAR440234 in patients with AML, ALL and MDS	SAR440234	No	I/II	R/R AML	DLT, toxicity, ORR, DOR, EFS	77	2022	Sanofi	USA, Germany, Spain	Recruiting
NCT03541369	CD135	Safety, tolerability, PK, PD, and efficacy of AMG 427 in subjects with R/R AML (20170528)	AMG 427	No	I	R/R AML	DLT, TEAEs, TRAEs	70	2022	Amgen	USA, Australia, Canada	Recruiting
NCT03038230	CLL-1	A phase I, multinational study of MCLA-117 in AML	MCLA-117	No	I	R/R AML, newly diagnosed elderly untreated AML	DLT, toxicity	50	2018	Merus N.V.	Belgium, France, Italy, Netherlands	Recruiting

#### Targeting CLL-1/CLEC12A with T-cell-recruiting antibody constructs

MCLA-117 [38] is a modified full-length human bispecific IgG and is the only CLEC12A x CD3 bispecific antibody currently in a clinical trial in adult patients with AML (NCT03038230). The target antigen, C-type lectin domain family 12 member A (CLEC12a, also named CLL-1), is expressed in the majority of AML cases, including on LSCs, but has not been detected on healthy HSCs, making it an attractive immunotherapeutic target [39–41]. The administration of MCLA-117 includes ramp-up dosing steps followed by weekly infusion at the target dose (each cycle is 20 days). Mascarenhas et al. reported preliminary results of this trial at the 2020 EHA Congress [42]. As of November 30, 2019, 50 patients had been treated with MCLA-117 with a target dose from 0.675 to 120 mg. No DLTs were identified. The most common TRAEs included pyrexia (32%), CRS (32%), chills (22%), infusion site phlebitis (14%), vomiting (12%), and nausea (10%). Grade 3 and 4 TRAEs included CRS (2%) and elevated liver transaminase (8%). Among 26 evaluable patients, four showed ≥50% blast reduction in the bone marrow, including one MLFS [42].

#### Targeting FLT3 with T-cell-recruiting antibody constructs

Like CLL-1, FLT3 (CD135) shows favorable expression in AML, with high expression intensities on bulk AML cells and LSCs, and low expression on healthy HSCs. AMG 427 is a CD3 x FLT3 half-life-extended BiTE [43]. In ex vivo experiments, the killing of AML cells by AMG 427 correlated with high FLT3 cell-surface levels and high (>1:38) E:T ratios, and was enhanced by the presence of an anti-PD-1 antibody [43]. AMG 427 is being evaluated in a phase I clinical trial in adults with R/R AML (NCT03541369).

#### Future directions

All of the bispecific antibodies used to treat AML are still in early clinical trials. As illustrated above, clinical data is available mainly in peer-reviewed abstracts and meeting presentations because these trials are still ongoing. Nonetheless, these preliminary results indicate that the safety profile of these bispecific antibodies is acceptable and suggest that bispecific antibodies might be promising therapeutics for treating AML.

There remain many unanswered questions. The most suitable antigens and, more specifically, the most appropriate epitopes of these antigens to target are yet to be identified. Unsurprisingly, CRS has been a common TRAE reported from the emerging data. Intriguingly, unlike with blinatumomab, neurotoxicity was not common among these reports. By using anti-inflammatory prophylaxis alone or in combination with a dose-step approach, high doses of bispecific antibodies were safely administered to patients. However, whether there are more convenient ways of administering bispecific antibodies while further reducing toxicity and improving efficacy remains to be investigated. In addition, the clinically most useful formats of bispecific antibodies remain undefined. Smaller formats have shorter in vivo half-lives, which, if necessary, makes interrupting or adjusting doses easier, but pose logistical challenges for dosing patients owing to the need for continuous infusion. Larger formats in general have slower clearance, thus longer in vivo half-lives meaning they cannot be shut off quickly but do not require continuous infusion. Moreover, larger formats that include Fc fragments can engage Fc-mediated cell killing, which might increase their efficacy [18, 19].

As T-cell function is key for the activity of bispecific antibodies, T-cell exhaustion might contribute to primary or secondary resistance. Knaus et al. [44] demonstrated a decrease in T-cell function in AML patients compared to healthy controls. All current AML BiTE trials are currently being investigated in R/R AML patients with a median of ≥4 prior treatment lines or post-allo-HSCT relapse (e.g., in the AMG 330 trial, up to 50% of patients had prior allo-HSCT). Moving forward, we believe that bispecific antibodies should be tested in earlier treatment lines including salvage-1 or even more optimally in the MRD setting, when there is likely to be an active and harnessable anti-AML T-cell immunity. As has been reported for BCP-ALL, in vivo and in preclinical models ex vivo in AML, that PD-L1 upregulation on AML cells is a common adaptive immune escape strategy [45]. The use of combination strategies of bispecifics with anti-PD-1 and anti-PD-L1 antibodies might help overcome such resistance and may be even more potent in earlier treatment lines with a better-preserved functional T-cell compartment. A study of AMG 330 with the PD-1 inhibitor pembrolizumab (NCT04478695) will evaluate this approach.

## Chimeric antigen receptor T-cell therapy

In contrast to bispecific antibodies, which transiently direct the patient’s endogenous T cells against target expressing cells, CAR-T cells are genetically modified autologous T cells equipped with a synthetic target-antigen receptor (the CAR) that expand after transfusion in a target-antigen dependent matter (so-called “living drug”). They have the potential to persist after infusion and induce a long-term antileukemic memory. The binding between a CAR and its antigen on a tumor cell triggers a signal transduction cascade through signaling domains that then activate T cells to kill the target either directly or by harnessing other components of the immune system [46]. CARs bind to their tumor antigens in an MHC-independent manner, which is their main advantage over regular TCRs [47].

Anti-CD19 CAR-T-cell therapies against B-lineage malignancies have been successfully used in clinical practice and are approved in the US and Europe [6]. In contrast to lymphoid malignancies, most AML antigens targeted by ADCs, bispecific antibodies, and CAR-T cells are frequently expressed in normal HSPCs or healthy organ tissues (e.g., liver, lung), increasing the risk of on-target, off-tumor toxicity. Accordingly, most clinical trials are currently applying CAR-T cell therapy as a “bridge to transplant” strategy, aiming at the eradication of chemorefractory (residual) AML cells to reduce relapse rates post-allo-HSCT while avoiding the risk of profound and prolonged cytopenia.

Early-phase AML CAR-T and CAR NK clinical trials, targeting CD33, CD123, and NKG2D are ongoing (Table 6). In a phase I study (NCT03018405, still recruiting) [48], 12 patients with hematological malignancies (eight AML, three MM and one MDS) received CYAD-01, a CAR product based on the receptor NKG2D with specificity for a broad range of ligands (MICA, MICB, and ULBP1-6) expressed on most tumors. CYAD-01 was administered without prior preconditioning therapy. CRS occurred in five patients, three at grade 1/2 and two at grade 3, and rapidly resolved with appropriate therapy such as tocilizumab. No neurotoxicity was observed. Of eight R/R AML patients, with a median of three prior therapies, seven were evaluable for response. The CR/CRi rate was 42% (three of seven patients, respectively). One patient proceeded to allo-HSCT and has been in durable response for more than 1 year.

**Table 6:** Selected ongoing trials of CAR-T cells therapy in AML.

Target	Phase	Study population	Intervention	Status	NCT.gov identifier
CD33	Phase I/II	Children and young adults with R/R AML	CD33 CAR-T cells	Recruiting	NCT03971799
CD33/CLL-1	Phase I	R/R high-risk hematologic malignancies	CD33/CLL-1 cCAR T cells	Recruiting	NCT03795779
CD123/CLL-1	Phase II/III	R/R AML	CD123/CLL-1 CAR-T cells	Recruiting	NCT03631576
CD123	Phase I	R/R AML	allogeneic anti-CD123 CAR-T cells (UCART123)	Recruiting	NCT03190278
CD123	Phase I	R/R AML after allo-HSCT	CD123CAR-41BB-CD3zeta-EGFRt-expressing T cells	Recruiting	NCT03114670
CD123	Phase I	CD123+ R/R AML and persistent/recurrent BPDCN	Autologous or allogeneic CD123CAR-CD28-CD3zeta-EGFRt-expressing T cells	Recruiting	NCT02159495
CD123	Phase I/II	R/R AML	CD123 CAR-T cells	Recruiting	NCT04272125
Muc1/CLL-1/CD33/CD38/CD56/CD123	Phase I/II	R/R AML	Muc1/CLL-1/CD33/CD38/CD56/CD123-specific gene-engineered T cells	Recruiting	NCT03222674
NKG2D	Phase I/II	Seven refractory cancers including AML	NKG2D CAR-T cells	Recruiting	NCT03018405
CD19	Phase I/II	CD19+ R/R AML	CD19 CAR-T cells	Recruiting	NCT03896854
CLL-1, CD33 and/or CD123	Phase I/II	R/R AML	CLL-1, CD33 and/or CD123-specific CAR gene-engineered T cells	Recruiting	NCT04010877
CD44v6	Phase I/II	R/R AML or MM expressing CD44v6	CD44v6 CAR-T cells	Recruiting	NCT04097301

Autologous CD123-specific CAR-T cells are under investigation (NCT02159495) for R/R AML (cohort 1) and blastic plasmacytoid dendritic cell neoplasm (BPDCN; cohort 2). Prior to T-cell infusion, all patients undergo lymphodepletion (fludarabine 25–30 mg/m^2^ for 3 days and cyclophosphamide 300 mg/m^2^ for 3 days). Patients receive a single dose of CD123-CAR-T cells with an option for a second infusion if they continue to meet safety and eligibility criteria and have persistent CD123+ disease at the end of cycle 1. At the most recent update [49], seven patients (six AML, one BPDCN) had received CD123-CAR-T cells. All six patients in the AML cohort had refractory AML following allo-HSCT, and a median of four (range 4–7) prior lines of therapy. One patient achieved CR and proceeded to a second allo-HSCT. Another patient with CR prior to treatment remained in CR post-therapy and proceeded to allo-HSCT. Two patients had blast reduction, including one patient who achieved MLFS. CRS occurred in five patients (four grade 1, one grade 2). All toxicities were reversible and manageable. There were no treatment-related cytopenias. In the BPDCN cohort, one patient with a bulky subcutaneous mass who did not respond to prior CD123 ADC therapy achieved CR after a single dose of CD123-CAR-T cells and continued in CR 60 days post-infusion. That patient tolerated the treatment well with no CRS or neurologic toxicity.

To overcome AML heterogeneity and the lack of tumor-specific antigen, and to mitigate toxicity due to the antigens common to leukemic blasts and normal tissues, dual-targeting CAR-T targeting strategies are being investigated [14]. In a phase I study, Liu et al. [50, 51] evaluated compound CAR (cCAR) T cells targeting two AML antigens, CD33 and CLL-1. The CLL-1b-CD33b cCAR consists of two individually complete and functional CAR molecules on the surface of a T-cell connected by P2A, a self-cleaving peptide linker. The study was designed with a CD52 safety switch. Patients received lymphodepletion with fludarabine and cyclophosphamide. To date, two unique responders have been reported from this trial [50, 51]; both had R/R AML treated with multiple lines of chemotherapy. Both patients had blast counts >20% before cCAR T-cell infusion, and both achieved MRD-negative remission and were able to proceed to allo-HSCT. The study is ongoing (NCT03795779).

Another novel strategy recently published by He et al. [52] was the isolation of multiple nanobodies (heavy-chain-only antibodies with a small single variable domain) that bind to various epitopes. By using a sequentially tumor-selected antibody and antigen retrieval (STAR) system, they developed a bispecific and split CAR (BissCAR) targeting CD13 and TIM3. This BissCAR T-cell effectively eradicated patient-derived AML with limited toxicity to normal HSCs, cells of myeloid lineage, and healthy organ systems in murine and patient-derived xenograft models [52]. This might be a promising approach for developing an effective CAR-T cell therapy for AML.

Despite the lack of an ideal AML antigen, concerns over CRS, and the potential for prolonged myelosuppression, the field of CAR-T cells as a therapeutic option in AML continues to make progress, both pre-clinically and clinically. Indeed, several strategies, such as gene-editing technology, combination therapies with checkpoint inhibitors or agonists, and targeting low-burden disease or MRD, are the subjects of early investigations to optimize CAR-T cells in AML. Specifically, genetic ablation of the CD33 antigen using CRISPR–Cas9 technology in human HSPCs has already been shown to be feasible, with multilineage hematopoietic recovery in an in vivo model system [53]. A first-in-human trial will be initiated that combines an allo-HSCT utilizing genetically modified, CD33-negative HSCs with CD33-directed CAR-T cells [54]. Advances in technology, in conjunction with AML-specific target-antigen identification, might allow CAR-T cell therapy in patients ineligible for allo-HSCT. However, this concept should be evaluated in a clinical setting before definitive recommendations can be made.

## Reactivation of endogenous T-cell responses against AML: immune checkpoint inhibitors

Immune checkpoints play an important role in the regulation of immune homeostasis by optimally balancing the stimulatory and inhibitory signals that mediate the T-cell immune response via co-stimulatory receptors such as CD28, OX40, CD27, and ICOS (expressed on T cells), or CD80 and CD86 (expressed on APCs), and co-inhibitory receptors, such as cytotoxic T-lymphocyte-associated protein 4 (CTLA-4) and programmed cell-death protein 1 (PD-1; both expressed predominantly, but not exclusively, on T cells) [55, 56]. ICPIs are approved in the United States and Europe for several solid tumors [57, 58]. In hematological malignancies, ICPIs are yet to be as widely developed or approved, although nivolumab and pembrolizumab are notable exceptions for the treatment of Hodgkin’s lymphoma and PMBCL [9, 59]. In AML, bone-marrow-infiltrating T-cell populations are preserved and may even be increased compared with bone marrows from healthy individuals, with an increased frequency of immune inhibitory and activating co-receptor expression (especially in relapsed AML), including PD-1, OX40, TIM3, and LAG3, suggesting a potential role for T-cell-harnessing therapies in AML [60–62]. Within the last five years, several ICPIs have been evaluated in clinical trials in patients with AML (Table 7) [56, 62].

**Table 7:** Ongoing clinical trials of checkpoint inhibitors in AML.

Setting	Checkpoint target	Intervention	Study design	Study population	Status	NCT.gov identifier
Single agent	PD-1 and CTLA-4	Nivolumab, ipilimumab	Phase I Three cohorts • Nivolumab • Ipilimumab • Nivolumab + ipilimumab	High-risk or R/R AML and MDS following allo-HSCT	Recruiting	NCT03600155
	PD-1 CTLA-4	Nivolumab, ipilimumab	Phase I Three cohorts • Nivolumab ipilimumab • Nivolumab + ipilimumab	AML and MDS at high risk of relapse post-allo-HSCT	Recruiting	NCT02846376
	PD-1 CTLA-4	Nivolumab, ipilimumab	Phase I Single cohort • Nivolumab or ipilimumab	Relapsed hematologic malignancies (including AML) post-allo-HSCT	Active, not recruiting	NCT01822509
	PD-1	Nivolumab	Phase I Two cohorts • Nivolumab post-HLA-matched unrelated donor HSCT • Nivolumab post-HLA-haploidentical donor HSCT	High-risk patients with MDS and AML post-allo-HSCT with post-transplant cyclophosphamide	Recruiting	NCT04361058
	PD-1	Nivolumab	Phase II Single cohort • Nivolumab	AML in remission at high risk for relapse	Recruiting	NCT02532231
	PD-1	Nivolumab	Randomized phase II Two cohorts • Nivolumab observation	AML patients in first complete remission after chemotherapy	Active, not recruiting	NCT02275533
	PD-1	Pembrolizumab	Phase I Single cohort • Pembrolizumab	AML, ALL or MDS with post-transplant relapse	Recruiting	NCT03286114
	PD-1	Pembrolizumab	Pilot study Single cohort • Pembrolizumab	AML, MDS, or mature B-cell lymphomas that have relapsed following allo-HSCT	Recruiting	NCT02981914
	PD-1	Pembrolizumab	Phase II Single cohort • Pembrolizumab	AML in remission not eligible for allo-HSCT	Active, not recruiting	NCT02708641
Combination with HMA	CTLA-4	Ipilimumab, decitabine	Phase I Two cohorts • Decitabine + ipilimumab for relapse post-allo-HSCT • Decitabine + ipilimumab for relapse in transplant-naïve patient	R/R AML or MDS	Recruiting	NCT02890329
	PD-1 CTLA-4	Nivolumab, ipilimumab, azacitidine	Phase II, nonrandomized Two cohorts • Azacitidine + nivolumab • Azacitidine + nivolumab + ipilimumab	Patients with R/R or newly diagnosed AML	Recruiting	NCT02397720
	PD-1	Pembrolizumab, decitabine	Phase I Two cohorts • Pembrolizumab + decitabine for patients with AML • Pembrolizumab + decitabine for patients with MDS	Patients with R/R or newly diagnosed AML or MDS	Not yet recruiting	NCT03969446
	PD-1	Nivolumab, azacitidine	Phase I Single cohort • Azacitidine + nivolumab post-HSCT	AML and high-risk myelodysplasia following reduce intensity allogeneic PBSC	Recruiting	NCT04128020
	PDL-1	Durvalumab, azacitidine	Randomized phase II Two cohorts • Azacitidine + durvalumab • Azacitidine alone	Higher-risk MDS or in elderly (≥65 years) AML subjects not eligible for HSCT	Active, not recruiting	NCT02775903
Combination with cytotoxic chemotherapy	PD-1	Pembrolizumab, conventional intensive chemotherapy	Randomized phase II Two cohorts • Conventional intensive chemotherapy + pembrolizumab • Conventional intensive chemotherapy	Newly diagnosed AML eligible for intensive induction chemotherapy	Not yet recruiting	NCT04214249
	PD-1	Nivolumab, idarubicin, cytarabine	Phase II • Single cohort • Idarubicin + cytarabine + nivolumab	High-risk AML and MDS	Active, not recruiting	NCT02464657
Other combination	PD-1	Ivosidenib (AG-120), nivolumab	Phase II Single cohort • Ivosidenib + nivolumab	Patients with IDH1-mutated R/R AML and high-risk MDS	Recruiting	NCT04044209
	PD-1	Pembrolizumab, azacitidine, venetoclax	Randomized phase II Two cohorts • Azacitidine + VEN • Azacitidine + VEN + pembrolizumab	Older patients with AML who are ineligible or refuse intensive chemotherapy	Not yet recruiting	NCT04284787
	PD-1	Nivolumab, decitabine, venetoclax	Phase I Single cohort • Nivolumab + decitabine + venetoclax	TP53-mutated AML	Recruiting	NCT04277442
	PDL-1	Atezolizumab, gilteritinib	Phase I/II Single cohort • Atezolizumab + gilteritinib	R/R FLT3-mutated AML	Active, not recruiting	NCT03730012
	PD-1	PDR001, decitabine MBG453	Randomized phase I Five cohorts • Decitabine + PDR001 • Decitabine + MBG453 • Decitabine + PDR001 + MBG453 • MBG453 + PDR001 • MBG453 alone	Patients with R/R AML or high-risk MDSD	Recruiting	NCT03066648
	CTLA-4	Ipilimumab CD25/Treg-depleted DLI	Phase I Single cohort • CD25/Treg-depleted DLI + ipilimumab	Myeloid disease relapse after matched HSCT	Recruiting	NCT03912064
	PDL- 1	Atezolizumab Hu5F9-G4	Phase I Single cohort • Atezolizumab + Hu5F9-G4	R/R FLT3-mutated AML	Recruiting	NCT03922477

## Single-agent checkpoint inhibitors and combinatorial approaches with hypomethylating agents

ICPIs have demonstrated very modest clinical efficacy as single agents in patients with R/R AML and myelodysplastic syndrome (MDS) who have not undergone allo-HSCT [63, 64].

Investigators have demonstrated that patients with AML/MDS treated with hypomethylating agents (HMAs) had dose-dependent upregulation of the surface expression of ICPI receptors and ligands (PD-L1, PD-L2, PD-1, and, to a lesser extent, CTLA-4) [65, 66]. Patients who had the highest PD-L1 upregulation had the shortest duration of response to HMA therapy, and a trend to inferior OS. This led to the hypothesis that the activation and upregulation of immune checkpoints during HMA therapy could be a possible mechanism of resistance, which might be overcome by combining HMA therapy with ICPIs [67, 68].

Nivolumab, an anti-PD-1 antibody, was combined with azacitidine in patients with R/R AML in a phase II clinical trial (NCT02397720) [69]. Among 70 patients treated, the ORR was 33%, including 22% with CR or CRi. Notably, this was a high-risk population, with 44% of the patients having secondary AML with poor risk cytogenetics, and a median of 2 (range 1–7) prior therapies. Grade 3/4 immune-related adverse events occurred in 11% of the patients, the most frequent being pneumonitis. The median OS for the 70 patients was 6.3 months, and among salvage-1 patients (*n* = 32) the median OS was 10.5 months. Patients with pre-therapy increased bone marrow CD3 and CD8 infiltration had a higher response rate, suggesting pre-therapy T-cell infiltration might be an indicator of an “inflamed tumor” and a biomarker for selecting patients likely to benefit from ICPI-based therapies. The anti-CTLA-4 antibody ipilimumab was added to the azacitidine and nivolumab backbone regimen in an ongoing expansion cohort of this phase II trial [70]. Twenty-four evaluable R/R AML patients were treated with the combination of azacitidine, nivolumab, and ipilimumab. The ORR was 44%, including 36% CR/CRi. Grade 3/4 immune-mediated toxicities were observed in six patients (25%), including rash, pneumonitis, and colitis.

In another phase II study (NCT02845297) the anti-PD-1 antibody pembrolizumab was given in combination with azacitidine to patients with R/R and newly diagnosed AML [71]. In the R/R AML cohort, four out of 29 patients (14%) evaluable for response achieved CR or CRi, and one (4%) PR. The median OS for the R/R AML cohort was 10.8 months. In the second cohort, 22 newly diagnosed AML older patients who were not candidates for intensive chemotherapy were enrolled. Among 17 evaluable patients, 47% achieved CR/CRi and 12% PR. The median OS for the frontline cohort was 13.1 months. Grade 3/4 immune-related adverse events were observed in nine patients (24%) in cohort 1, and three patients (14%) in cohort 2.

The results of a phase II, randomized, international, multicenter study (NCT02775903) of azacitidine with or without the PD-L1 antibody durvalumab in frontline therapy for high-risk MDS (cohort 1) or AML (cohort 2) were recently reported [72]. Cohort 2 randomized (1:1) 129 AML patients ≥65 years old ineligible for intensive chemotherapy. There were no statistically significant differences in the ORR (31.3% vs. 35.4%) or CR rate (17.2% vs. 21.5%) between azacitidine with durvalumab versus azacitidine alone. The median OS for azacitidine with durvalumab and azacitidine alone was 13.0 and 14.4 months, respectively. No concerning or unexpected safety signals were noted. Notably, more than 50% of the patients discontinued the trial medications and were censored for survival analysis, which might impact the interpretation of the results.

Interestingly, PD-1 inhibition and PD-L1 inhibition appear to have differential efficacy profiles in AML and MDS, as has been shown in solid tumors [73]. Herbrich et al. [74] evaluated bone marrow and peripheral blood samples by single-cell mass cytometry (CYTOF) profiling of serially collected samples from nine R/R AML patients treated with azacitidine and PD-L1 inhibitor avelumab (NCT02953561). Four of the nine evaluable patients experienced an initial blast reduction and seven had subsequent rapid disease progression. Serial measurements from the same patients were used to phenotypically track both resistant and newly emerging clones. Whereas PD-L1 levels were consistently low in baseline and on-treatment sample analyses, the seven who developed initial blast reduction followed by rapid progression exhibited high PD-L2 protein expression on AML cells. PD-L2 was also frequently expressed in emerging clones not present at baseline. According to the authors, this observation suggests that PD-1 and PD-L1 inhibition might not be the same in AML and might help explain, at least in part, the apparent discrepancy in response rates and survival with PD-1 inhibitor- versus PD-L1 inhibitor-based therapies seen in the clinical trials in patients with AML. Ongoing analysis using CYTOF and single-cell RNA sequencing should help us better understand the mechanistic differences between PD-1- and PD-L1-based therapies [73].

## Checkpoint inhibitors in combination with cytotoxic chemotherapy

Chemotherapy may augment the immune response against cancer. In vivo experiments in mouse models have shown that the injection of cytosine arabinoside (cytarabine) induced the expression of CD80 and CD86, and reduced the expression of PD-1 on leukemic cells, making them more susceptible to cytotoxic T-lymphocyte-mediated killing [75]. In a phase II study, nivolumab was combined with idarubicin and cytarabine in patients with newly diagnosed AML or high-risk MDS (>10% blasts) [76]. Forty-four patients were enrolled, of whom 42 had AML and two had MDS. The ORR was 78%, comprising 64% complete responses and 14% CRi. Of these 34 responders, 18 proceeded to allo-HSCT. At a median follow-up of 17.3 months, the median OS for all patients was 18.5 months. Six patients had grade 3/4 immune-related adverse events. The combination was deemed safe with no concerning toxicities pre- or post-allo-HSCT. These results, did not, in the opinion of the authors, demonstrate clear improvement in CR rates, MRD negativity rates, EFS, or OS over standard therapies in this population, and the study was terminated.

In the R/R setting, a phase II trial examined high-dose cytarabine followed by pembrolizumab [77]. Thirty-seven patients with R/R AML received age-adjusted high-dose cytarabine followed by pembrolizumab 200 mg IV administered on day 14 of the cytarabine. The ORR (CR + CRi + PR + MLFS) was 46% and the CR/CRi rate was 38%. Nine patients (24%) proceeded to allo-HSCT. There were no instances of grade >3 acute GvHD or veno-occlusive disease post-allo-HSCT. At a median follow-up of 7.8 months, the median OS was 8.9 months. This study is ongoing.

## Immune checkpoint inhibitors in minimal residual disease and maintenance

Eradication of MRD is an active area of investigation in AML therapy. Preclinical data suggests that immune checkpoint pathways might contribute to tumor persistence by enabling leukemic cells to escape immune surveillance [78]. In a mouse AML model with MRD positivity, persisting leukemic blasts became more resistant over time to cytotoxic T-cell-mediated killing, concomitantly associated with increased PD-L1 and CTLA-4 expression. Blocking this pathway in vitro and in vivo prolonged the survival of the mice [78]. NCT02532231 is an ongoing single-arm phase II study of nivolumab as a maintenance therapy for patients with high-risk AML in CR who are ineligible for allo-HSCT. Fourteen patients were enrolled [79]. High-risk features were five (36%) with persistent MRD, four (29%) with adverse cytogenetics, one (7%) adverse mutation alone, one t-AML (7%) and three patients (21%) in ≥CR2. Seventy-one percent of patients were in CR at 12 months, which is encouraging. This study is ongoing. NCT02275533 is a randomized phase II study investigating the role of nivolumab in eliminating MRD in patients with AML in CR after completion of the planned chemotherapy.

## Future directions

ICPIs appear to have clinical activity in AML, albeit with less impressive results than in patients with solid tumors and certain lymphomas. Several reasons for this have been proposed, including the heterogenicity of AML with diverse clonality and multiple driver mutations [80], as well as the relatively lower mutational burden in AML cells, thereby possibly limiting the repertoire of leukemia-specific antigens available to prime the T-cell response [80–82]. The protective bone-marrow microenvironment might also exert an immunosuppressive influence by preventing access of T cells to AML blasts or by secretion of immune-dampening metabolites such as indoleamine 2,3-dioxygenase, 2-hydroxyglutarate, and arginine by the AML blasts [83].

Combining ICPIs with another AML therapy might improve their activity, as mentioned above. As multiple targeted and signaling therapies have recently been approved for AML, ongoing trials are combining ICPIs with these new backbone regimens. NCT04277442 is a phase I trial combining nivolumab with decitabine and venetoclax in frontline TP53-mutated AML, NCT02397720 is evaluating nivolumab with azacitidine and venetoclax in R/R and frontline AML, and NCT04284787 is a phase II trial of pembrolizumab in combination with azacitidine and venetoclax in newly diagnosed AML patients deemed unsuitable for induction therapy. NCT03730012 is a phase I/II trial evaluating atezolizumab with gilteritinib in R/R FLT3-mutated AML, and NCT04044209 is evaluating nivolumab with ivosidenib (AG-120) in R/R IDH1-mutated AML.

Pertinent questions on treatment stratification will hopefully be answered with ongoing clinical trials. Identifying biomarkers that will help select patients most likely to benefit from ICPIs is of the highest importance for choosing the optimal setting (frontline, MRD+, maintenance, early salvage) and the ideal combination partners and/or sequence to improve outcomes while maintaining an acceptable safety profile.

## Conclusions

The last 3–5 years have seen significant progress made in the understanding of the immune biology of AML [84] and advances in technology resulting in the development of novel AML-directed T-cell therapeutic approaches. Despite the numerous ongoing trials, we believe that T-cell immunotherapies for myeloid malignancies are still in their infancy. Such trials will help AML immunotherapeutics evolve and advance in the coming years. We predict that these clinical advances will be accelerated by a focused analysis of biomarkers at the pre-therapy, on-therapy, and relapse stages. These data can help us to identify the patients most likely to respond, to elucidate the mechanisms of immune resistance/escape [85], validate novel checkpoints and AML-specific targets, and better manage immune toxicities. In addition to biomarker-driven strategies, identifying and deploying these therapies in optimal clinical settings such as MRD, low-burden disease, and early salvage will be important. The application of novel techniques such as single-cell RNA and DNA sequencing, single-cell cytokine analysis, and mass cytometry on patient samples, to unravel at a granular level the role of the tumor microenvironment and non-T-cell compartments in immune response or resistance is likely to add critical information to guide combinatorial or sequential immune therapy approaches. Thus, we can look forward to an exciting and hopefully fruitful next decade for immunotherapies for AML.
